# Supplementary material for: Fast and accurate estimation of the covariance between pairwise maximum likelihood distances
Source: PeerJ. 2014 Sep 25;2:e583. doi: 10.7717/peerj.583 (PMC4179615; doi:10.7717/peerj.583)
Supplement: Supplemental Information 1 — (1) The MAPLE code supporting the proof in Section “r-state symmetric model”; (2) the MAPLE code deriving equations (31)–(33); (3) Figures S1 and S2. [file peerj-02-583-s001.pdf]

Supplemental Material
for
Fast and Accurate Estimation of the Covariance between Pairwise Maximum Likelihood Distances

Manuel Gil

S.1 Maple code for derivation in Section *r-state symmetric model*

In this section we give a Maple program which follows the derivation presented in Section *r-state symmetric model*. In particular, the code evaluates the expression on the right-hand side of Equation 16. At the end it compares the derived expression with the conjectured one and demonstrates their equivalence.

```
pm := B*(1-exp(-d/B)): r := 1/(1-B):
Pr_Quartet := proc (i, j, k, l, m)
global r, pm:
    `if`(i = 0, 1-subs(d = ei, pm), subs(d = ei, pm)/(r-1)) *
    `if`(k = 0, 1-subs(d = ek, pm), subs(d = ek, pm)/(r-1)) *
    `if`(m = 0, 1-subs(d = em, pm), subs(d = em, pm)/(r-1)) *
    `if`(j = 0, 1-subs(d = ej, pm), subs(d = ej, pm)/(r-1)) *
    `if`(l = 0, 1-subs(d = el, pm), subs(d = el, pm)/(r-1))/r
end:
f0 := r*(r-1):
f1 := r*(r-1)^2:                    #   \ ei          ej /
f2 := r*(r-1)*(r-2):                #    \      em      /
f3 := r*(r-1)^2*(r-2):              #     o------------o
f4 := r*(r-1)*(r-2)^2:              #    /              \
f5 := r*(r-1)^2*(r-2)^2:            #   / ek          el \
f6 := r*(r-1)*((r-2)^2+r-1):
f7 := r*(r-1)*((r-2)^2+r-1)*(r-2):
f8 := r*(r-1)*((r-2)^2+r-1)^2:
PAT := [[[0, 0, 0, 0, 1], f0],
       [[0, 1, 0, 1, 0], f1], [[1, 0, 1, 0, 0], f1],
       [[0, 1, 1, 0, 0], f1], [[1, 0, 0, 1, 0], f1],
       [[0, 0, 0, 1, 1], f2], [[0, 0, 1, 0, 1], f2],
       [[0, 1, 0, 0, 1], f2], [[1, 0, 0, 0, 1], f2],
```

```
        [[0, 1, 1, 1, 0], f3], [[1, 0, 1, 1, 0], f3],
        [[1, 1, 0, 1, 0], f3], [[1, 1, 1, 0, 0], f3],
        [[0, 1, 0, 1, 1], f4], [[1, 0, 1, 0, 1], f4],
        [[0, 1, 1, 0, 1], f4], [[1, 0, 0, 1, 1], f4],
        [[1, 1, 1, 1, 0], f5], [[0, 0, 1, 1, 1], f6],
        [[1, 1, 0, 0, 1], f6], [[1, 1, 0, 1, 1], f7],
        [[1, 1, 1, 0, 1], f7], [[0, 1, 1, 1, 1], f7],
        [[1, 0, 1, 1, 1], f7], [[1, 1, 1, 1, 1], f8]]:
E_SijSkl := 0:
for p in PAT do E_SijSkl := E_SijSkl+Pr_Quartet(op(p[1]), pm)*p[2] od:
E_SijSkl := simplify(E_SijSkl):
E_Sij := subs(d = ei+em+ej, pm): E_Skl := subs(d = ek+em+el, pm):
cov_IijIkl := (E_SijSkl-E_Sij*E_Skl)*n:
hatd := solve(pm = I_/n, d):
Dhatd_DI := simplify(subs(I_ = n*pm, diff(hatd, I_))):
Dhatd_DI_ij := subs(d = ei+em+ej, Dhatd_DI):
Dhatd_DI_kl := subs(d = ek+em+el, Dhatd_DI):
cov := expand(Dhatd_DI_ij*Dhatd_DI_kl*cov_IijIkl):
cov_conjecture := B*((1-B)*exp(2*em/B)+(2*B-1)*exp(em/B)-B)/n:
proven := evalb(expand(cov_conjecture-cov) = 0); # evaluates to "true"
```

S.2 Maple code for weighted least squares on a quartet

The method of weighted least squares for phylogenetic tree reconstruction from pairwise distance data was first proposed by Cavalli-Sforza and Edwards [1], and Fitch and Margoliash [2]. The goal is to find an unrooted tree T (topology and branch lengths) that minimizes

$$S(T) = \sum_{i,j} \frac{(t_{ij}(T) - d_{ij})^2}{v_{ij}}, \tag{1}$$

where t_{ij} is the path length on T between leafs i and j, d_{ij} the corresponding input distance and v_{ij} its variance. Sometimes the variances are not known and, therefore, modeled as a function of the distance estimates. For instance, Fitch and Margoliash assumed that the variances are proportional to the squared distances. When nothing is known about the errors, or if they are assumed to be independently distributed and equal for all observed distances, then all the variances are set to one. This leads to the ordinary least squares method.

The minimization of S is a non-trivial problem. It involves searching the discrete space of unrooted binary tree topologies whose size is exponential in the number of leaves n. The minimization for a given topology is a linear least squares problem formulated by the normal equations. Their algebraic solution involves the computation of a pseudo-inverse. However, elegant combinatorial formulas have been derived for certain variance-structures [3].

In Section *Topological relation and path length* we consider quartet trees. In this case there are only three topological relations so that the enumeration of

the tree space is not a problem. We will derive closed form solutions for the direct computation of S and the corresponding optimal branch lengths. The approach implemented in the following Maple script is to take the derivatives of S with respect to the branch lengths $\{e_i\}_{i=1}^{5}$, equate each of the derivatives to zero, solve the resulting system of equations, and substitute the optimized branch lengths $\{\hat{e}_i\}_{i=1}^{5}$ in S:

```
S :=   (e1+e2   -d12)^2/v12       #   \ e1          e3 /
     + (e1+e5+e3-d13)^2/v13       #    \      e5      /
     + (e1+e5+e4-d14)^2/v14       #     o------------o
     + (e2+e5+e3-d23)^2/v23       #    /              \
     + (e2+e5+e4-d24)^2/v24       #   / e2          e4 \
     + (e3+e4   -d34)^2/v34:
dS1 := diff(S,e1): dS2 := diff(S,e2): dS3 := diff(S,e3):
dS4 := diff(S,e4): dS5 := diff(S,e5):
hat_e := solve({dS1=0, dS2=0, dS3=0, dS4=0, dS5=0},
               {e1,e2,e3,e4,e5}):
Sopt := simplify(subs(hat_e,S));
hat_e5 := simplify(rhs(hat_e[5]));
```

The output of the script corresponds to Equations 29 and 30.

S.3 Supplemental Figure

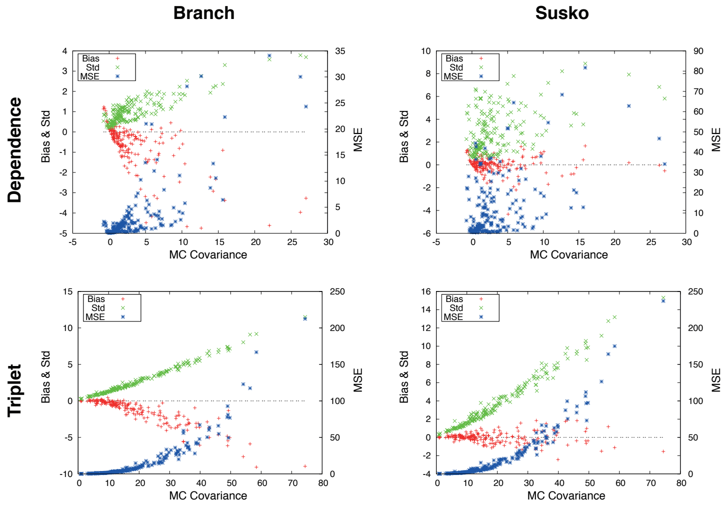

Figure 1: Ungapped simulation. Bias (red, left y-axis), standard deviation (green, left y-axis), and mean squared error (blue, right y-axis) of the branch-covariance (first row) and the Susko-covariance (second row) for the dependence (first column) and triplet case (second column) as a function of the Monte Carlo covariance for sequences of length 500 amino-acids.

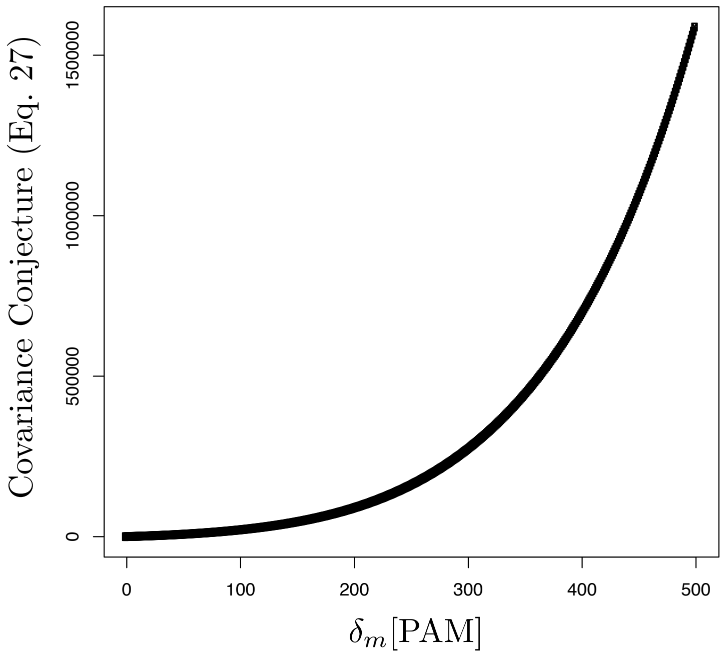

Figure 2: Covariance conjectured in Equation 27 as a function of the shared path length δ_m for the GCB model from 0 to 500 PAM with sequence-length $n = 1$. (The sequence-length is a constant factor in Equation 27.)

References

[1] L. L. Cavalli-Sforza and A. W. F. Edwards. Phylogenetic analysis: Models and estimation procedures. *Evolution*, 21:550–570, 1967.

[2] W.M. Fitch and E. Margoliash. The construction of phylogenetic trees. *Science*, 155:279 – 284, 1967.

[3] R Mihaescu and L Pachter. Combinatorics of least-squares trees. *PNAS*, 105(36):13206–13211, 2008.
